# Pregabalin as an effective treatment for acute postoperative pain following spinal surgery without major side effects: protocol for a prospective, randomized controlled, double-blinded trial

**DOI:** 10.1186/s13063-023-07438-2

**Published:** 2023-06-22

**Authors:** Ki-Hoon Park, Nam-Su Chung, Hee-Woong Chung, Tae Young Kim, Han-Dong Lee

**Affiliations:** Department of Orthopaedic Surgery, Ajou University School of Medicine, Suwon, South Korea

**Keywords:** Spinal surgery, Postoperative pain, Gabapentinoid, Pregabalin

## Abstract

**Background:**

Patients experience considerable postoperative pain after spinal surgery. As the spine is located at the centre of the body and supports body weight, severe postoperative pain hinders upper body elevation and gait which can lead to various complications, including pulmonary deterioration and pressure sores. It is important to effectively control postoperative pain to prevent such complications. Gabapentinoids are widely used as preemptive multimodal analgesia, but their effects and side effects are dose-dependent. This study was designed to examine the efficacy and side effects of varying doses of postoperative pregabalin for the treatment of postoperative pain after spinal surgery.

**Methods:**

This is a prospective, randomized controlled, double-blind study. A total of 132 participants will be randomly assigned to the placebo (*n* = 33) group or to the pregabalin 25 mg (*n* = 33), 50 mg (*n* = 33), or 75 mg (*n* = 33) groups. Each participant will be administered placebo or pregabalin once prior to surgery and every 12 h after surgery for 72 h. The primary outcome will be the visual analogue scale pain score, total dose of administered intravenous patient-controlled analgesia, and frequency of rescue analgesic administered for 72 h from arrival to the general ward after surgery, subdivided into four periods: 1–6 h, 6–24 h, 24–48 h, and 48–72 h. The secondary outcomes will be the incidence and frequency of nausea and vomiting due to intravenous patient-controlled analgesia. Safety will be assessed by monitoring the occurrence of side effects such as sedation, dizziness, headache, visual disturbance, and swelling.

**Discussion:**

Pregabalin is already widely used as preemptive analgesia and, unlike nonsteroidal anti-inflammatory drugs, is not associated with a risk of nonunion after spinal surgery. A recent meta-analysis demonstrated the analgesic efficacy and opioid-sparing effect of gabapentinoids with significantly decreased risks of nausea, vomiting, and pruritus. This study will provide evidence for the optimal dosage of pregabalin for the treatment of postoperative pain after spinal surgery.

**Trial registration:**

ClinicalTrials.gov NCT05478382. Registered on 26 July 2022.

**Supplementary Information:**

The online version contains supplementary material available at 10.1186/s13063-023-07438-2.

## Administrative information

**Table:** 

Title {1}	Pregabalin as an effective treatment for acute postoperative pain following spinal surgery without major side effects: protocol for a prospective, randomized controlled, double-blinded trial
Trial registration {2a and 2b}	The trial was registered with the ClinicalTrials.gov with the National Clinical Trial (NCT) number: NCT05478382
Protocol version {3}	11 October 2022, version number 1.4
Funding {4}	External funding from HK inno.N Corp.(Seoul, Korea)
Author details {5a}	1. Ki-Hoon Park, MD: Department of Orthopaedic Surgery, Ajou University School of Medicine, Suwon, South Korea, kihoon05@naver.com 2. Nam-Su Chung, MD: Department of Orthopaedic Surgery, Ajou University School of Medicine, Suwon, South Korea, namsuchung@gmail.com 3. Hee-Woong Chung, MD: Department of Orthopaedic Surgery, Ajou University School of Medicine, Suwon, South Korea, life04ung@gmail.com 4. Tae-Hyoung Kim, MD: Department of Orthopaedic Surgery, Ajou University School of Medicine, Suwon, South Korea, ajouosresident2@gmail.com 5. *Han-Dong Lee, MD: Department of Orthopaedic Surgery, Ajou University School of Medicine, Suwon, South Korea, handonglee@gmail.com *Corresponding author
Name and contact information for the trial sponsor {5b}	HK inno.N Corp.: Ji Su Kim, jisu.kim@inno-n.com, 100, Eulji-ro, Jung-gu, Seoul, Republic of Korea
Role of sponsor {5c}	The sponsor and funding source does not have a role in the design; collection, management, analysis, and interpretation of data; writing of the report; and the decision to submit the this or other manuscripts for publication

## Introduction

### Background and rationale

Postoperative pain after spinal surgery is debilitating for the patients due to its severe intensity. As the spine is located at the centre of the body to support body weight, severe postoperative pain hinders upper body elevation and gait, which can lead to various complications, including pulmonary deterioration and pressure sores [1, 2]. Effective control of postoperative pain is important for the prevention of such complications and for fast rehabilitation [3, 4].

Preemptive multimodal analgesia is often performed using acetaminophen and nonsteroidal anti-inflammatory drugs (NSAID) [5, 6]. However, NSAIDs have a risk of complications, such as renal toxicity, bleeding tendency, and cardiac events, limiting their use in the elderly or in patients with pre-existing renal insufficiency [7, 8]. NSAID use following spinal fusion has also been reported to increase the rate of nonunion [9]. In contrast, gabapentinoids including gabapentin and pregabalin are relatively safe for patients with renal or cardiac risk factors, do not hinder fusion after spinal surgery, show opioid-sparing effects, and decrease the adverse effects of intravenous patient-controlled analgesia (IV PCA) [10, 11]. Due to these advantages and their efficacy, gabapentinoids are widely used for the management of postoperative pain after various surgeries [12–14].

Pregabalin binds to the α2-δ protein subunit of presynaptic voltage-gated calcium channels in both the central and peripheral nervous systems, and it has a superior pharmacodynamic and pharmacokinetic profile to that of gabapentin [15]. It is a more potent and more effective analogue of gabapentin [16]. However, gabapentinoids are associated with several adverse effects, including sedation, dizziness, and peripheral oedema, which are known to be dose-dependent [17]. There is currently some controversy regarding the optimal safe dose of gabapentinoids.

### Objectives

The objective of this study is to examine the efficacy and safety of varying doses of pregabalin for the treatment of postoperative pain after spinal surgery.

### Trial design

This will be a single-centre, randomized, prospective, double-blind, superiority clinical trial with four arms. The trial will include three treatment arms where pregabalin will be administered at different doses (25, 50, 75 mg), and one control arm where placebo will be administered. The participants will be equally distributed among the four arms.

## Methods: participants, interventions, and outcomes

### Study setting

This trial will take place at Ajou University Hospital in Suwon, South Korea. The trial protocol has been approved by the Institutional Review Boards of our institution (AJOUIRB-CT-2022–350). This study was registered at ClinicalTrials.gov (National Clinical Trial number: NCT05478382), and the protocol was designed following the guidelines of the Consolidated Standards of Reporting Trials and Standard Protocol Items: Recommendations for Interventional Trials (SPIRIT) (Additional file 1). We plan to begin trial recruitment in October 2022, and the planned study period will be 4 years. A total of 132 participants will be enrolled after recruitment is announced, and allocation will be performed by stratified block randomization.

### Eligibility criteria

Patients undergoing spinal surgery for degenerative lumbar disorders will be recruited from the Department of Orthopaedic Surgery, at Ajou University Hospital. Identified patients will be approached for inclusion in the study based on the inclusion/exclusion criteria described below.

Inclusion criteria are as follows: age 19 years or older, patients undergoing spinal surgery for degenerative lumbar disorders, and voluntary agreement to participate in the study after receiving sufficient explanation.

Exclusion criteria are as follows: minors under the age of 19 years, patients assigned to American Society of Anesthesiologists classification class 3–6, previous frequent dizziness or headaches, active alcohol or drug usage, having taken any analgesics daily or within 48 h before surgery, impaired renal (glomerular filtration rate < 60 mL/min/1.73 m^2^) and/or hepatic function (Child Pugh classification B and C), diagnosed with and treatment for anxiety or depressive disorders, and coverage from worker’s compensation insurance or car insurance.

Patients with severe adverse reactions to our drug of interest, such as dizziness, drowsiness, or peripheral oedema, will be excluded. Patients with any postoperative complications including infection, nerve injury, hematoma, metal implant-related problems, and death, will also be excluded.

### Who will take informed consent?

During the preoperative visit, on arrival at the hospital department the day before surgery, one of the trial members who has a medical doctor’s license will explain the study protocol and obtain written informed consent from the patients or their guardians. On the day of surgery, the investigating physician will collect the signed consent form after ensuring that the patient has understood the information leaflet and recheck the inclusion and exclusion criteria.

### Additional consent provisions for collection and use of participant data and biological specimens

There are no plans for the use of participants’ data and biological specimens in ancillary studies.

## Interventions

### Explanation for choice of comparators

Patients will be randomly assigned to one of four groups: pregabalin 25 mg, 50 mg, 75 mg, or placebo. Placebo was chosen as an appropriate comparator because the intervention is proposed as an adjunct to routine postoperative pain control. Currently, there are no recommendations for routine perioperative analgesia.

### Intervention description

Patients will be allocated into one of four groups: placebo group (P0), pregabalin (Kabalin; HK inno.N Corp., Seoul, South Korea) 25 mg group (P25), pregabalin 50 mg group (P50), and pregabalin 75 mg group (P75) [18]. The P0 group will take three placebo capsules once prior to surgery and every 12 h after surgery for 72 h. The P25 group will take one 25 mg pregabalin capsule and two placebo capsules, the P50 group will take two 25 mg pregabalin capsules and one placebo capsule, and the P75 group will take three 25 mg pregabalin capsules, all according to the same schedule. The 25 mg pregabalin capsule and placebo capsule will have the same appearance for blinding.

### Criteria for discontinuing or modifying allocated interventions

Participants who present with severe drowsiness, dizziness, visual disturbance, or swelling that precludes the use of pregabalin will discontinue the trial and stop taking their allocated treatment. Participants who develop infection, nerve injury, hematoma, or metal-related complications following surgery or those who request discontinuation will also be excluded from the trial.

### Strategies to improve adherence to interventions

Participants will be directly observed taking their medications by clinical nurses and clinical research associates (CRAs) at the study sites to encourage and monitor adherence.

### Relevant concomitant care permitted or prohibited during the trial

After the surgical procedure, IV PCA (PS-1000; Unimedics, Seoul, Korea) will be administered and continued for 72 h. The PCA regimen will consist of fentanyl 1500 μg (total volume including saline: 150 mL) and will be administered at 2 mL/h as background infusion and 2 mL on demand with a 30-min lockout.

Concomitant therapy for comorbidities, such as hypertension, diabetes, rheumatoid arthritis, etc., will be permitted and documented. Any analgesic use is prohibited from 48 h before surgery until 72 h after surgery. If the participant asks for additional pain control, rescue analgesics, including oxycodone (IRcodon tab 5 mg; Unimed, Seoul, Korea), fentanyl patch (Durogesic patch 12 μg/h; Janssen Korea, Seoul, Korea), or pethidine (Pethidine 25 mg injection; Hana Pharm Co., Seoul, Korea), will be administered.

If participants present with nausea or vomiting, ramosetron (Nasea Injection 0.3 mg/2 ml; Daiichi Sankyo, Seoul, Korea) will be administered intravenously. If symptoms continue after ramosetron administration, metoclopramide (Macperan Injection 10 mg/2 ml; Dong Wha Pharm Co., Seoul, Korea) will be administered intravenously.

### Provisions for post-trial care

The principal investigator has insurance, in accordance with the legal requirements in South Korea, that provides coverage for any injuries or deaths caused by the study. The insurance covers the injury/death occurring during the study or within 1 year after study completion.

### Outcomes

We will examine the efficacy of pregabalin on postoperative pain using the variables described in the literature [12]. The primary outcomes are pain intensity measured on a visual analogue scale (VAS) (length, mm), total administered amount of IV PCA measured (volume, mL), and frequency of rescue analgesics administered (the number of administrations, according to the type of rescue analgesic, and the total amount of morphine equivalent dose). The VAS score is evaluated using a pain ruler marked from 0 (no pain) to 100 (maximal pain) mm, with patients directly indicating their pain level. The secondary variables are the incidence and frequency of nausea and vomiting caused by IV PCA. Assessments of all variables will be performed during the first 72 h following the arrival of the participant at the general ward, subdivided into four periods: 1–6 h, 6–24 h, 24–48 h, and 48–72 h. We will evaluate pain between the midpoint and the end of each of the four time zones, aiming to measure as close to the end time as possible and use the pain scores at all four time points as the primary outcome. The pain levels in each time zone will be compared to see if they differ depending on the treatment method. Through this, we will ascertain how the treatment effect changes over time and how these changes differ among different treatment groups. Through these variables, we plan to check the effects of pregabalin on reduction of postoperative pain and IV PCA usage. In addition, we will examine whether reduction of IV PCA usage has a beneficial effect on the patient by reducing the incidences of common side effects, nausea and vomiting.

### Participant timeline

**Fig. 1:**
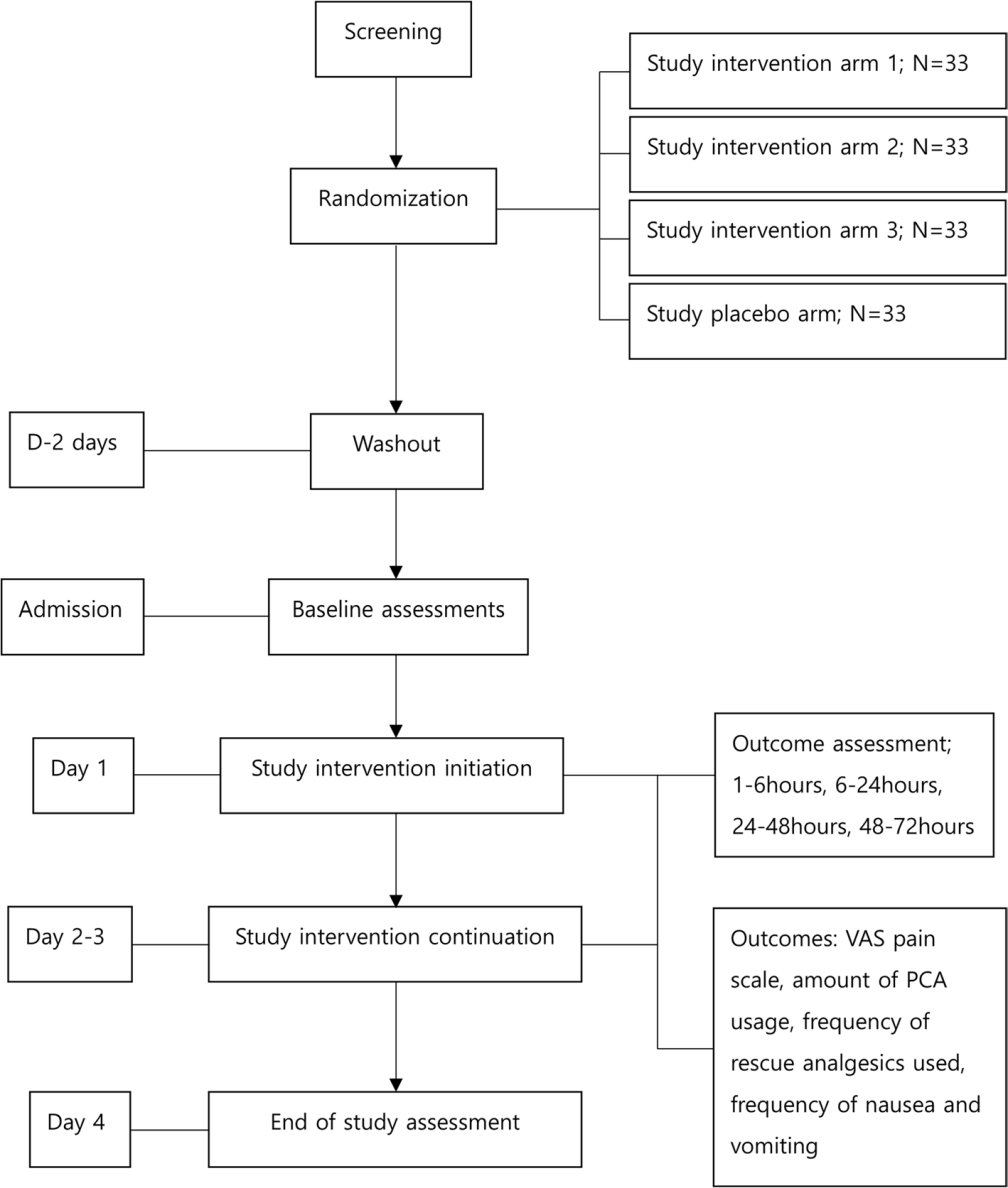
Schematic diagram showing the timeline for key procedures

The participant timeline is shown in Fig. 1.

### Sample size

The prior calculation of the sample size indicated that at least 28 subjects were required for each group. For calculation, the software G*Power 3.1.9.7 was used with a statistical power of 0.95 at an effect size of 0.448 with an alpha level of 0.0167. The effect size was determined based on a previous study [18]. In this study, three groups—those treated with placebo, pregabalin 75 mg, and pregabalin 150 mg—each comprised 28 participants. The groups were compared based on their PCA usage, with mean values of 95.3, 96.6, and 105.2 respectively, and an approximate standard deviation of 10. For the purposes of this study, which involves four instead of three groups, a fourth hypothetical group was considered with the mean value of 96.6, which is the median value of the three established groups. Based on this data, Cohen’s *f* was calculated, resulting in an effect size of 0.448. To compare the four groups through Bonferroni’s test, the alpha level was set at 0.05 divided by 4, which is 0.0167. When calculated based on these parameters, the total sample size needed is found to be 112, and the number of participants needed per group is 28. Considering a follow-up loss of approximately 15%, 33 participants per group would be required, resulting in a total of 132 participants needed for the study.

### Recruitment

Participants will be recruited from among patients visiting our outpatient clinic, so no other efforts to recruit potential subjects are required. Patients will receive a detailed explanation of the risks and benefits of the trial to ensure that they fully understand the specifics of the study, and then they will be asked to voluntarily provide a signed informed consent form.

## Assignment of interventions: allocation

### Sequence generation

All patients will be randomized to one of the following four groups at a 1:1:1:1 ratio one day prior to surgery: P0 (*n* = 33), P25 (*n* = 33), P50 (*n* = 33), and P75 (*n* = 33) groups. The assignment will be conducted by computer-generated random sequence with block randomization, stratified by sex and age (< 65 and ≥ 65 years). Based on the potential risk of age and gender having an impact on the pain relief and side effect outcomes after surgery, as suggested by previous literature, we performed stratification accordingly [19]. To maintain an equal allocation of participants to each of the four treatment groups, we will use a block size of 4 (one allocation for each treatment group within a block). The generated sequence will be masked from the primary investigators and will be recorded in the randomization list.

### Concealment mechanism

The allocation sequence will be concealed using sealed, opaque, and stapled envelopes and will not be opened by the investigators involved in the management or assessment of the study participants. After the allocation, trial participants, care providers, and outcome assessors will remain blinded throughout the trial. The placebo will have the same appearance, route of administration, and dosing schedule as those of the study drug to maintain participant blinding. Neither the names of the study drugs nor the study arm will be included in the medication packs.

### Implementation

The allocation sequence will be generated by a member of the study staff not involved in the care of the participants, outcome assessment, or analysis of the unblinded data. The unblinded staff member in charge of the allocation will deliver the concealed sequence to the pharmacy department of our institute, where the unblinded pharmacist will provide the participants with their allocated study drug based on the randomized sequence. The unblinded staff and pharmacist are not involved in the management or assessment of the study participants.

## Assignment of interventions: blinding

### Who will be blinded

The participants and investigators will remain blinded to the group allocation until the trial has ended.

### Procedure for unblinding if needed

Emergency unblinding of the patient allocation will be performed if patients meet the following criteria: severe violation of the treatment plan; severe adverse reaction to the study drug, including drowsiness, dizziness, and peripheral oedema; request by the patient or their family members to stop the trial; perioperative complications, including infection, vascular injury, hematoma, and prosthesis-related complications; and death during the trial.

## Data collection and management

### Plans for assessment and collection of outcomes

Data will be collected using case report forms (CRFs) on paper, which will be completed by trained CRAs from days 1 to 4. The schedule of assessments is shown in Table 2.

**Table 2:** Schedule of patient enrolment, interventions, and assessments

**Time point**	Study period							
	Enrolment	Allocation	Post-allocation					
	D-14	D0	Surgery	1 h	6 h	24 h	48 h	72 h
**Enrolment:**	X							
Eligibility screen	X							
Informed consent	X							
Inclusion and exclusion criteria	X							
Allocation		X						
**Interventions:**								
Pregabalin				X	X	X	X	X
Placebo				X	X	X	X	X
**Assessments:**								
VAS score				X	X	X	X	X
IV PCA usage				X	X	X	X	X
Frequency of rescue analgesic use				X	X	X	X	X
Frequency of nausea and vomiting				X	X	X	X	X
Sedation				X	X	X	X	X
Dizziness				X	X	X	X	X
Headache				X	X	X	X	X
Visual disturbance				X	X	X	X	X
Swelling				X	X	X	X	X

### Plans to promote participant retention and complete follow-up

The intervention will occur during the first 72 h after surgery and will be monitored closely by the study team throughout that period. Evaluation of postoperative pain using the VAS and postoperative complications are part of the routine postoperative patient examination. Patients do not need to adhere to any other specific tasks, and there is no outpatient clinic follow-up as part of the study, so there will be no difficulties in completing the evaluation.

### Data management

Data for screening will be recorded by the CRAs. After enrolment, all study data will be recorded into the CRFs on paper. Medical history will be obtained by interviewing the participants. Data will be entered into a computer for analysis by H-W Chung and K-H Park. Data will be stored for 3 years after the end of the study. Data accuracy will be checked by the principal investigator of the study.

### Confidentiality

Patients will be referred to exclusively by their participant numbers. Clinical information, informed consent, and CRFs will be stored separately within a double locker until expiration of the storage period, after which the data will be destroyed.

### Plans for collection, laboratory evaluation, and storage of biological specimens for genetic or molecular analysis in this trial/future use

This trial does not involve collection of biological specimens.

## Statistical methods

### Statistical methods for primary and secondary outcomes

The primary objective of this study is to compare the effect of different doses of pregabalin to placebo on pain relief and the need for additional analgesics during the 72-h period following surgery, at various time points. Additionally, the study aims to assess the incidence of adverse events during this period. Primary outcomes include the VAS pain score, total dose of IV PCA administered, and frequency of rescue analgesic administered. We will use the Kolmogorov–Smirnov test (K-S test) to assess the normality of the primary outcome measure. If the normality assumption is met, we will perform an analysis of variance (ANOVA) analysis; otherwise, we will use the Kruskal–Wallis test for comparison. We will use the Bonferroni’s method for conducting subgroup analysis to account for multiple comparisons. Categorical variables, such as the incidence and frequency of nausea and vomiting caused by IV PCA or the incidence of side effects (sedation, dizziness, headache, visual disturbance, and swelling), will be compared between the groups using the chi-squared test. Statistical analyses will be performed using SPSS 20.0 (IBM, Armonk, NY, USA). In all analyses, *P* < 0.05 will be taken to indicate statistical significance.

### Interim analysis

We have no plans to perform or publish interim analyses.

### Methods for additional analyses (e.g. subgroup analyses)

No additional analyses are planned.

### Methods in analysis to handle protocol non-adherence and any statistical methods to handle missing data

We analyse the data of all patients who meet the inclusion and exclusion criteria, complete the study without dropout within 72 h after surgery. In case of missing data during the study, patients will be excluded. In the case of dropping out from the study, no further data will be collected; however, the exact reason for withdrawal will be documented. We expect a withdrawal rate of 20% per group.

### Plans to give access to the full protocol, participant-level data, and statistical code

Access to participant-level deidentified data and statistical data will be considered by the corresponding author upon reasonable request.

## Oversight and monitoring

### Composition of the coordinating centre and trial steering committee

The coordinating centre is composed of clinicians at the Department of Anesthesiology and Pain Medicine and the Department of Orthopaedic Surgery of Ajou University Hospital, including the principal investigator. The centre consists of the principal investigator (HDL) and two investigators (KHP and HWC) who are responsible for recruiting the patients and conducting the study. The trial steering committee will check and discuss whether the study is being conducted appropriately the day after enrolment of each patient.

### Composition of the data monitoring committee, its role and reporting structure

In this study, the research team determined that an independent data monitoring committee is not necessary, as there will be no interim analysis, patients involved have non-critical conditions and will undergo treatment for a relatively brief period, and the drug under investigation has a well-characterized safety profile with no known harm to patients. Furthermore, due to the brief intervention duration and limited sample size, no interim analysis will be conducted.

### Adverse event reporting and harms

During the intervention, the principal investigator will actively seek and be informed of any occurring adverse events. All adverse events will be recorded in the CRF. The principal investigator will report any serious adverse events to the institutional review board within 15 days of notice. Adverse events that are not serious will be reported every 3 months. When reporting adverse events or harm, the causality, timing of occurrence, severity, seriousness, and provided management will be reported together.

### Frequency and plans for auditing trial conduct

In this study, audits will be performed annually, or as deemed necessary based on the study’s progress and any identified issues by the institution’s quality assurance team. The inspections will be independent of those of the investigators and the sponsor. The auditor will review essential study documents, such as the study protocol, informed consent forms, case report forms, data management plans, and monitoring reports, to assess compliance with Good Clinical Practice and regulatory requirements. The auditor will also check the accuracy and completeness of the data collected during the trial, comparing source documents (e.g. medical records) with case report forms to ensure consistency and identify any discrepancies or missing data.

### Plans for communicating important protocol amendments to relevant parties (e.g. trial participants, ethical committees)

Although protocol amendments are not expected, any deviations from the protocol will be fully documented in a breach report form, reported to all regulatory bodies, and thoroughly recorded in a protocol deviation log. Protocol amendments will be submitted first to the sponsor within 7 days and then to the relevant parties by sending the updated protocol to the investigators. A copy of the revised protocol will be added to the Investigator Site File. The protocol will also be updated on the ClinicalTrials.gov registry website.

### Dissemination plans

The findings from this study will be disseminated via local and international conference presentations, as well as publication in peer-reviewed scientific journals.

## Discussion

This is a single-centre, randomized, prospective, double-blind clinical trial designed to provide evidence on the efficacy and side effects of varying doses, especially low doses, of pregabalin for the treatment of postoperative pain after spinal surgery. To our knowledge, there have been few studies regarding the optimal dose and side effects of postoperative pregabalin for the management of postoperative pain after spinal surgery. Although this study will be conducted at a single centre, the results may still be generalizable to a wider population. The study design, including the inclusion and exclusion criteria, and the use of randomized controlled trials help to minimize bias and increase the internal validity of the study. Additionally, the study population may be representative of the larger population in terms of demographics, health status, and other relevant factors.

Pregabalin binds to the α2-δ subunit of presynaptic voltage-gated calcium channels in both the central and peripheral nervous systems, reducing excitatory transmitter release and spinal sensitization [20]. Pregabalin binds to the subunit more strongly than gabapentin and shows a clear dose–response relationship and high absorption rate at all doses [21]. Moreover, pregabalin has been reported to show a better analgesic profile during the postoperative period, in comparison with gabapentin [16]. Due to these advantages, pregabalin is a key agent for the management of postoperative pain after spinal surgery and is already included in numerous guidelines, including Enhanced Recovery After Surgery for Spine Surgery and the Clinical Guidelines for Multidisciplinary Spine Care of the North American Spine Society [22, 23].

However, there is ongoing controversy regarding the efficacy of pregabalin for postoperative pain, with systemic reviews and meta-analyses yielding conflicting results. One systematic review reported that gabapentin and pregabalin were efficacious in reducing postoperative pain and narcotic requirements after lumbar spinal surgery [3]. Another randomized, double-blind, placebo-controlled study supported the clinical use of pregabalin in a postsurgical setting for pain relief [24]. In contrast, other studies reported that pregabalin did not show a clinically significant analgesic effect on postoperative pain [25–27].

The adverse effects of postoperative pregabalin have not been investigated in detail. Most studies focused on the efficacy and side effects of pregabalin when used preoperatively [25, 27]. Kim et al. [18] reported the incidence of pregabalin side effects until 48 h postoperatively, but the doses of pregabalin used in that study were 75 mg and 150 mg. Our study will focus on the efficacy and adverse effects of postoperative pregabalin at lower doses (25 mg, 50 mg, and 75 mg).

In conclusion, our study will provide evidence on the efficacy of pregabalin for postoperative pain relief after spinal surgery without major side effects even when used at the low dose of 25 mg.

## Trial status

Protocol version: v1.4 (date: October 11, 2022)

Recruitment of the first patient: expected to begin at October 2022

Expected date for completion of recruitment: May 31, 2024

## Supplementary Information

**Additional file 1.**

## Data Availability

After completion of this trial and publication of the results, data sharing will be considered upon reasonable request to the corresponding author.

## Abbreviations

VAS: Visual analogue scale
IV PCA: Intravenous patient-controlled analgesia
NSAID: Nonsteroidal anti-inflammatory drugs
SPIRIT: Standard Protocol Items: Recommendations for Interventional Trials
CRA: Clinical research associates
CRF: Case report form
